# Assessing frailty in older adults: discriminative properties of physical activity questionnaires

**DOI:** 10.3389/fpubh.2025.1702843

**Published:** 2025-12-05

**Authors:** Mert Doğan, Özge Erol Doğan, Fatma Ayvat, Ender Ayvat

**Affiliations:** 1 Department of Physiotherapy and Rehabilitation, Faculty of Health Sciences, Akdeniz University, Antalya, Türkiye; 2 Department of Elderly Care, Vocational School of Health, Ardahan University, Ardahan, Türkiye; 3 Department of Physiotherapy and Rehabilitation, Faculty of Physical Therapy and Rehabilitation, Hacettepe University, Ankara, Türkiye

**Keywords:** physical activity, frailty, assessment, physical activity scale for older adults, international physical activity scale, discriminative ability, cut-off value

## Abstract

**Introduction:**

Frailty is prevalent among older adults and is a growing public health concern. Physical activity questionnaires are pragmatic tools for screening; however, their discriminative capacity remains uncertain. This study aimed to determine the discriminative performance and optimal cut-offs of the International Physical Activity Questionnaire–Short Form (IPAQ-SF) and the Physical Activity Scale for the Elderly (PASE) for identifying frailty states (non-frail, pre-frail, and frail) in a clinically derived cohort of older adults.

**Methods:**

The study enrolled 289 older adults who were assessed using a demographic form, the FRAIL Scale, the IPAQ-SF, and the PASE. Frailty discrimination was evaluated via receiver operating characteristic analysis (ROC); optimal thresholds were identified using the Youden index, diagnostic indices (sensitivity, specificity, accuracy, predictive values) were computed, micro-/macro- area under the curves (AUC) were reported, and scale AUCs were compared using the DeLong test.

**Results:**

Participants had a mean age of 72.5 ± 7.0 years, and 56.7% were women. Frailty prevalence was 31.1% non-frail, 29.4% pre-frail, and 39.4% frail. For IPAQ-SF, AUCs were 0.45 (non-frail), 0.40 (pre-frail), and 0.67 (frail); for PASE, AUCs were 0.39 (non-frail), 0.50 (pre-frail), and 0.65 (frail). Macro−/micro-AUCs were 0.51/0.53 for IPAQ-SF and 0.51/0.52 for PASE. No significant differences were found between the two instruments across the frailty categories (DeLong test, *p* > 0.05). At optimal cut-offs (IPAQ-SF ≤ 322.5; PASE ≤ 63.6), both questionnaires showed moderate agreement with the FRAIL Scale (*κ* = 0.46–0.48), whereas agreement between the instruments was only low-to-moderate (*κ* = 0.32, McNemar *p* = 0.015).

**Conclusion:**

IPAQ-SF and PASE demonstrated limited ability to discriminate frailty status in older adults, with moderate accuracy in identifying frail individuals and poor discrimination between the non-frail and pre-frail groups. The IPAQ-SF showed slightly higher specificity, whereas the PASE demonstrated higher sensitivity, indicating that they capture distinct aspects of physical activity behavior but cannot be used interchangeably.

## 1 Introduction

Frailty is a common clinical syndrome among older adults, characterized by diminished physiological reserve and increased vulnerability to external stressors. With advancing age, progressive functional decline occurs across multiple physiological systems, reducing the body’s ability to maintain homeostasis and withstand stress. Consequently, older individuals become more susceptible to adverse outcomes, such as hospitalization, functional and cognitive decline, and even mortality (1–3).

Frailty is influenced by a broad range of biopsychosocial factors, including low educational attainment, inadequate nutrition, malnutrition, depression, low physical activity levels, polypharmacy, cancer, and dementia (4). As the global population ages, the prevalence of frailty continues to rise, posing substantial challenges to healthcare systems. Reported prevalence rates vary widely across regions, ranging from 7 to 12% in the United States, 7.8% in Hispanic populations, and 5.8–27% in Europe (5–8). These figures highlight frailty as a significant public health issue. Given its high prevalence and multifactorial nature, reliable and valid assessment methods are essential for early identification and timely interventions.

Various tools are currently used to assess frailty in older adults. The among the most commonly used are the Clinical Frailty Scale, the Pictorial Fit-Frailty Scale, the FRAIL Scale, the Edmonton Frail Scale, and the Tilburg Frailty Indicator (9, 10). However, owing the multidimensional nature of frailty, relying on a single measure may not fully capture the complexity of the syndrome. Therefore, complementary assessments such as handgrip strength, short- and long-distance walking tests, physical activity questionnaires, balance evaluations, cognitive assessments, and functional performance tests are recommended (11–13). Among these domains, physical activity has received particular attention because of its strong association with frailty and its modifiable nature.

Low levels of physical activity are considered one of the strongest and most modifiable risk factors for frailty in older adults. Prospective cohort studies and meta-analyses have consistently demonstrated that higher levels of physical activity significantly reduce the risk of frailty (14, 15). Among the available instruments, International Physical Activity Questionnaire–Short Form (IPAQ-SF) and the Physical Activity Scale for the Elderly (PASE) were selected because they are the most widely used, validated, and culturally adapted self-report tools for assessing physical activity in older populations. Both questionnaires have established Turkish versions with strong reliability and construct validity (16, 17). Moreover, they represent complementary measurement perspectives: while the IPAQ-SF quantifies activity intensity and energy expenditure using metabolic equivalents (METs), the PASE emphasizes age-relevant activities such as household and caregiving tasks that are critical for frailty assessment. Previous studies have applied these tools to frailty-related populations, demonstrating varying diagnostic accuracies depending on sample characteristics and analytical approaches (18, 19). Therefore, comparing their discriminative capacities within the same clinical cohort may provide valuable insights into their relative performance and suitability for frailty screening. In this context, physical activity questionnaires serve as practical, low-cost, and easily applicable tools for use in clinical and community settings. Nevertheless, their discriminative ability to accurately identify frailty remains insufficiently explored, highlighting the need for further investigation. To address this gap, the present study aimed to determine the optimal cut-off values of two widely used physical activity questionnaires for identifying frailty in older adults and to evaluate their sensitivity, specificity, accuracy, and diagnostic odds ratios.

## 2 Methods

### 2.1 Study design and participants

This cross-sectional study was conducted at the Faculty of Physical Therapy and Rehabilitation, Hacettepe University, following approval by the institutional ethics committee (Decision Number: FTREK24/92). Data collection was carried out between December 2024 and June 2025, with a total study duration of approximately 9 months.

Participants were primarily recruited through community-based announcements, including invitation posters distributed in public spaces. Additional participants were also recruited through the outpatient clinics of the faculty. A snowball sampling strategy was employed to recruit additional eligible participants.

The inclusion criteria were as follows:

- Aged 65 years or older,
- Ability to read, understand, and respond to study questionnaires,
- No history of severe neuropsychiatric or neurological disorders (e.g., dementia, Parkinson’s disease, schizophrenia) that could affect participation,
- No major communication or motor impairments that would prevent questionnaire completion,
- Mini-Mental State Examination (MMSE) score > 24,

Individuals who did not meet these criteria were excluded from the study. Written informed consent was obtained from all participants.

### 2.2 Data collection

Data were collected through face-to-face interviews lasting approximately 45 min. Each participant completed a general evaluation form, the FRAIL Scale, the International Physical Activity Questionnaire–Short Form (IPAQ-SF), and the Physical Activity Scale for the Elderly (PASE).

### 2.3 Measures

#### 2.3.1 General evaluation form

This form was developed by the research team to record demographic and clinical characteristics, including age, sex, height, weight, number of medications, and medical history.

#### 2.3.2 International physical activity questionnaire–short form (IPAQ-SF)

The IPAQ-SF is a widely used instrument designed to assess self-reported physical activity levels in various populations. It consists of seven items evaluating the frequency and duration of vigorous-intensity, moderate-intensity, and walking activities performed for at least 10 min during the previous week, as well as time spent sitting. Physical activity levels are expressed in MET-minutes/week using standardized values (vigorous: 8.0 MET, moderate: 4.0 MET, walking: 3.3 MET, sitting: 1.5 MET). The IPAQ-SF is short, easy to administer, and allows for international comparability, making it appropriate for large-scale population-based studies. Saglam et al. (17) established the Turkish validity and reliability of the scale (17, 20).

#### 2.3.3 Physical activity scale for the older adults (PASE)

The PASE was specifically developed for older adults to evaluate physical activity across three domains: leisure, household, and occupationial activities (19). It captures not only exercise but also age-relevant daily activities, such as gardening, housework, and caregiving, which are important for this population. Participants reported the frequency (never, rarely, sometimes, often) and duration (<1 h, 1–2 h, 2–4 h, >4 h) of activities performed during the previous week. Scores were calculated according to the PASE manual, with higher scores indicating higher levels of activity. The Turkish version was validated by Ayvat et al. (16) and Washburn et al. (21).

#### 2.3.4 FRAIL scale

The FRAIL Scale is a brief, five-item screening tool that assesses fatigue, resistance, ambulation, illness, and weight loss. Each item is scored as 0 or 1, yielding a total score ranging from 0 to 5. Participants are classified as non-frail (0 points), pre-frail (1–2 points), or frail (≥3 points). The FRAIL Scale is widely recommended as a simple and valid screening measure for frailty in both clinical and research settings. It was originally developed by Morley et al. (33) and validated in Turkish by Hymabaccus et al. (10, 22).

### 2.4 Statistical analysis

All statistical analyses were performed using MATLAB R2023b (MathWorks Natick, MA, USA). Continuous variables were summarized as mean ± standard deviation or median (interquartile range), and categorical variables as frequencies and percentages. To assess the discriminative capacity of the IPAQ-SF and PASE across frailty categories (non-frail, pre-frail, and frail), multiclass ROC curve analysis (one-vs-rest approach) was conducted. Given the inverse association between physical activity and frailty, questionnaire scores were mathematically inverted prior to analysis so that higher transformed values reflected a higher frailty likelihood. This allowed for a uniform interpretation of the AUC, sensitivity, and specificity across all models. The area under the curve (AUC) with 95% confidence intervals (DeLong and bootstrap methods) was calculated, and optimal cut-off points were determined using the Youden index. Diagnostic indices, including sensitivity, specificity, accuracy, predictive values, F1 score, Matthews correlation coefficient (MCC), and diagnostic odds ratio (DOR), were calculated. Precision–recall (PR) curves with AUC-PR were also reported. Macro- and micro-averaged AUCs were computed, and DeLong’s test was applied to compare the performances of IPAQ-SF and PASE. Agreement analysis was also performed. Cross-tabulations were generated to evaluate the classification agreement between the IPAQ-SF and PASE and between each questionnaire and the FRAIL Scale. Cohen’s kappa coefficient was calculated to quantify agreement strength, while McNemar’s test was used to assess systematic disagreement. A two-tailed *p*-value < 0.05 was considered statistically significant. The sensitivity of power estimates to clinically meaningful AUC differences (*Δ* = 0.10) was also evaluated.

## 3 Results

A total of 289 older adults participated in this study. The mean age of the participants was 72.5 ± 7.0 years, with 56.7% women and 43.3% men. The mean height and body weight were 162.5 ± 8.6 cm and 71.6 ± 12.4 kg, respectively. At least one chronic condition was present in 32% of the participants, and the mean number of medications used was 2.0 ± 1.6. Regarding physical activity, the mean PASE score was 66.7 ± 45.8, while the mean IPAQ total score was 731.8 ± 905.3 MET-min/week. Frailty status was distributed as follows: 31.1% non-frail (*n* = 90), 29.4% pre-frail (*n* = 85), and 39.4% frail (*n* = 114) (Table 1).

**Table 1:** Demographic and clinical characteristics of the participants.

Characteristics (*n* = 289)	Value [Mean ± SD/*n* (%)]
Age (years)	72.5 ± 7.0
Height (cm)	162.5 ± 8.6
Weight (kg)	71.6 ± 12.4
Sex	
Female	164 (56.7%)
Male	125 (43.3%)
Chronic disease	91 (32.0%)
Number of medications	2.0 ± 1.6
PASE score	66.7 ± 45.8
IPAQ-SF score (MET-min/week)	731.8 ± 905.3
Frailty status	
Non-frail	90 (31.1%)
Pre-frail	85 (29.4%)
Frail	114 (39.4%)

Data are presented as mean ± standard deviation (SD) or number (percentage). PASE, Physical Activity Scale for the Elderly; IPAQ-SF, International Physical Activity Questionnaire-Short Form; MET, Metabolic equivalent of task.

The ROC analyses yielded, for IPAQ-SF, AUCs of 0.45 (95% CI: 0.36–0.54) for non-frail, 0.40 (95% CI: 0.31–0.48) for pre-frail, and 0.67 (95% CI: 0.61–0.74) for frail. For PASE, the AUCs were 0.39 (95% CI: 0.29–0.49) for non-frail, 0.50 (95% CI: 0.40–0.59) for pre-frail, and 0.65 (95% CI: 0.58–0.72) for frail. The sensitivity, specificity, accuracy, PPV, NPV, and other diagnostic indices for both instruments are presented in Table 2.

**Table 2:** Diagnostic performance of physical activity scales (IPAQ-SF and PASE) for frailty classification: ROC analysis results.

Test	Comparison	AUC (95% CI)	AUCB (95% CI)	Optimal cut-off	Sensitivity	Specificity	Accuracy	PPV	NPV	F1-score	MCC	DOR
IPAQ-SF	Non-frail	0.45 (0.36–0.54)	0.45 (0.36–0.55)	4626.00	1.00	0.00	0.17	0.16	1.00	0.28	0.03	0.20
	Pre-frail	0.40 (0.31–0.48)	0.40 (0.31–0.49)	2199.00	0.95	0.09	0.20	0.14	0.92	0.24	0.05	1.82
	Frail	0.67 (0.61–0.74)	0.67 (0.62–0.74)	322.50	0.71	0.63	0.65	0.36	0.88	0.48	0.29	4.17
PASE	Non-frail	0.39 (0.29–0.49)	0.39 (0.30–0.49)	14.03	0.19	0.86	0.75	0.20	0.84	0.20	0.05	1.40
	Pre-frail	0.50 (0.40–0.59)	0.50 (0.41–0.59)	61.23	0.61	0.51	0.52	0.16	0.89	0.25	0.08	1.57
	Frail	0.65 (0.58–0.72)	0.65 (0.59–0.72)	63.60	0.79	0.54	0.60	0.34	0.90	0.47	0.27	4.33

AUC, area under the curve; AUCB, area under the curve with 5,000 Bootstrap; CI, confidence interval; PPV, positive predictive value; NPV, negative predictive value; MCC, Matthew’s correlation coefficient; DOR, diagnostic odds ratio.

Macro−/micro-ROC analyses demonstrated macro-AUC and micro-AUC values of 0.51 and 0.53 for IPAQ-SF, and 0.51 and 0.52 for PASE, respectively. The DeLong test indicated no statistically significant differences between the IPAQ-SF and PASE in the non-frail (*p* = 0.33), pre-frail (*p* = 0.08), or frail groups (*p* = 0.51). Similarly, the pooled micro-analysis revealed no significant difference between the two instruments (*p* = 0.92) (Table 3).

**Table 3:** Multiclass ROC analysis and comparisons of AUC values between IPAQ-SF and PASE.

Performance metrics	IPAQ-SF	PASE	*z*	*p*
Overall Performance				
Macro Average AUC	0.51	0.51	–	–
Micro Average AUC	0.53	0.52	–	–
AUCs Comparison by Group*				
Non-frail	0.45	0.39	0.97	0.33
Pre-frail	0.40	0.50	−1.73	0.08
Frail	0.67	0.65	0.66	0.51
Micro (pooled OvR)	0.53	0.52	0.10	0.92

^*^The statistical significance between the AUC values of IPAQ-SF and PASE was compared using the DeLong test. OvR, one vs rest.

Both instruments demonstrated moderate agreement with the FRAIL Scale in identifying frail individuals (IPAQ-SF: *κ* = 0.48; PASE: *κ* = 0.46). In contrast, the direct comparison between the IPAQ-SF and PASE yielded only low-to-moderate agreement (*κ* = 0.32), and the classification differences were statistically significant (McNemar *p* = 0.015) (Table 4).

**Table 4:** Cut-off based agreement between IPAQ-SF, PASE, and FRAIL scale classification.

Comparison	Cross-tab (% of total, *N* = 289)	Cohen’s Kappa	McNemar *p*-value
IPAQ-SF vs. PASE	Both Frail = 93 (32.2%); Both Non-frail = 97 (33.6%); Disagreement: IPAQ+ / PASE- = 21 (7.3%), IPAQ- / PASE+ = 78 (27.0%)	0.32 (low–moderate)	0.015
IPAQ-SF vs. FRAIL Scale	TP = 85 (29.4%); TN = 130 (45.0%); FP = 45 (15.6%); FN = 29 (10.0%)	0.48 (moderate)	–
PASE vs. FRAIL Scale	TP = 95 (32.9%); TN = 115 (39.8%); FP = 60 (20.8%); FN = 19 (6.6%)	0.46 (moderate)	–

Cut-off values were ≤322.5 for IPAQ-SF and ≤63.6 for PASE. Frail classification was defined by the FRAIL Scale. McNemar test was performed only for IPAQ-SF vs PASE to compare paired classifications.

**Figure 1:**
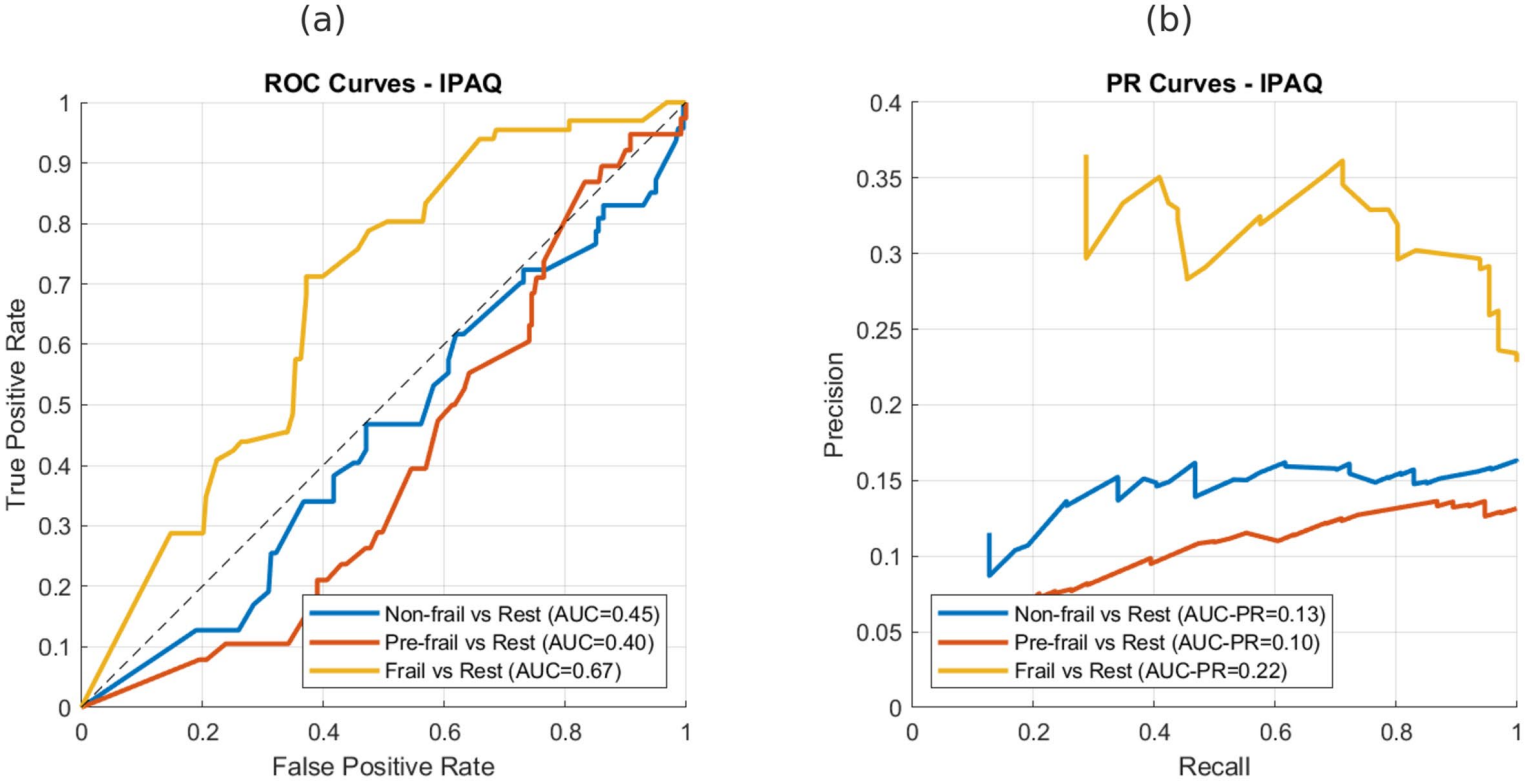
ROC and precision–recall curves for IPAQ-SF in discriminating frailty groups. **(a)** ROC curves for non-frail, pre-frail, and frail groups (AUC = 0.45, 0.40, and 0.67, respectively). **(b)** Precision–recall curves for non-frail, pre-frail, and frail groups (AUC-PR = 0.13, 0.10, and 0.22, respectively).

**Figure 2:**
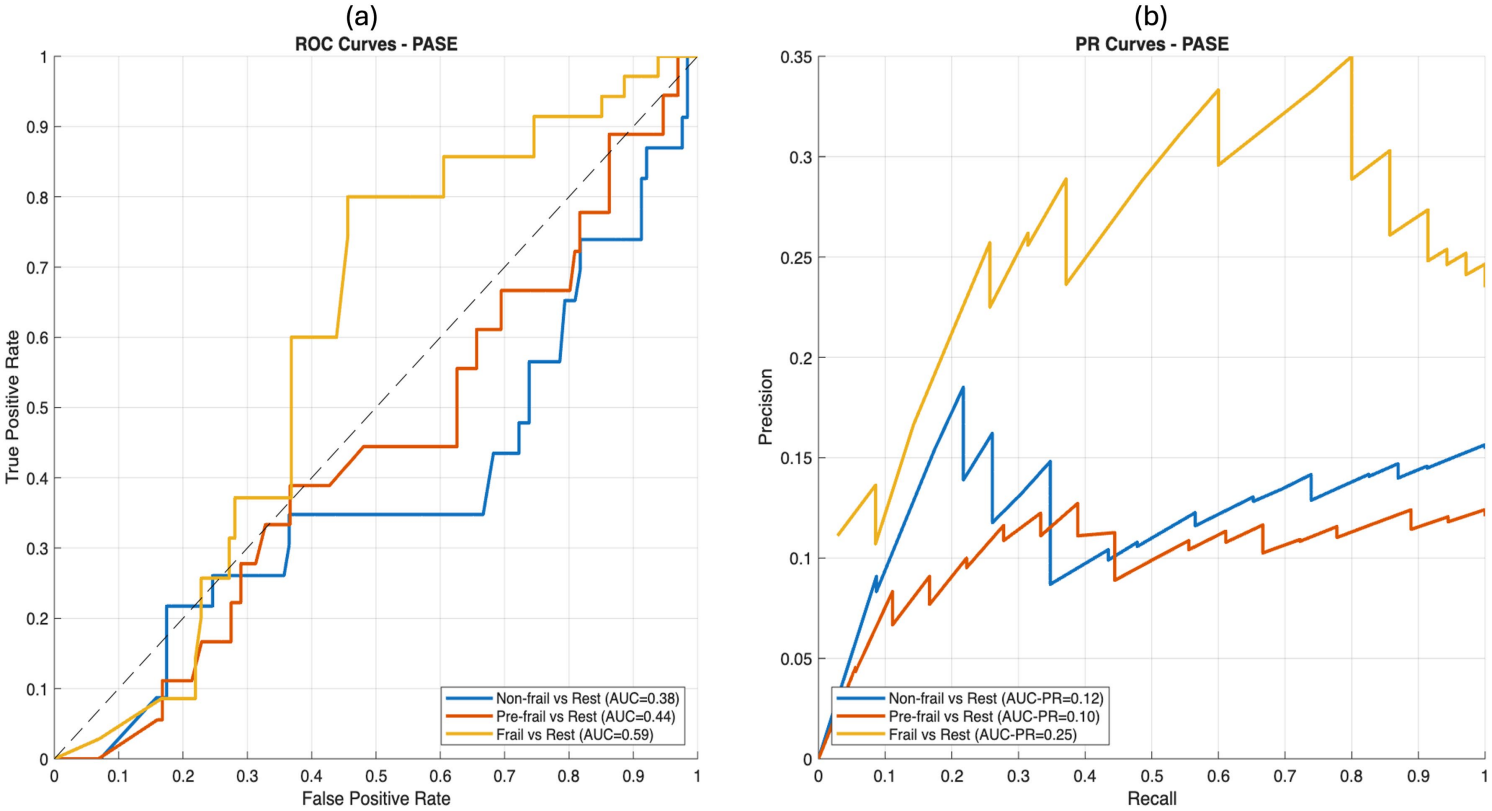
ROC and precision–recall curves for PASE in discriminating frailty groups. **(a)** ROC curves for non-frail, pre-frail, and frail groups (AUC = 0.39, 0.50, and 0.65, respectively) **(b)** Precision–recall curves for non-frail, pre-frail, and frail groups (AUC-PR = 0.13, 0.12, and 0.28, respectively).

Figures 1, 2 show the ROC and precision–recall curves for the IPAQ-SF and PASE, respectively. For IPAQ-SF (Figure 1), fair-to-moderate discrimination was observed in the frail group (AUC = 0.67), whereas discrimination for the non-frail and pre-frail groups was close to chance level.

A similar pattern was observed for the PASE (Figure 2), with an AUC of 0.65 in the frail group and poor discrimination in the non-frail and pre-frail groups. Precision–recall analyses further confirmed these findings, indicating that both instruments demonstrated limited ability to distinguish non-frail and pre-frail individuals while showing fair-to-moderate precision in identifying frail participants.

Post-hoc power analyses were performed using the method described by Hanley and McNeil (32), which estimates the variance of the area under the receiver operating characteristic (ROC) curve (AUC) based on the sample size and case–control distribution. For the frail group, the observed AUCs of 0.67 for IPAQ-SF and 0.65 for PASE were associated with post-hoc statistical powers of 1.00 and 0.99, respectively, at a two-sided *α* = 0.05. Sensitivity checks further confirmed ≥0.90 power for identifying a *Δ* = 0.10 increase over the chance AUC (AUC = 0.50) with the observed sample sizes. Post-hoc power analysis indicated adequate statistical power for detecting discrimination above the chance level.

Detailed subgroup analyses of diagnostic performance, including ROC metrics for sex- and age-based classifications, as well as comparative AUC statistics between the IPAQ-SF and PASE, are presented in Supplementary material S2.

## 4 Discussion

The present study evaluated the discriminative ability of two widely used physical activity questionnaires, the International Physical Activity Questionnaire–Short Form (IPAQ-SF) and the Physical Activity Scale for the Elderly (PASE), in identifying frailty in older adults. The main findings indicate that both instruments demonstrated only fair accuracy in discriminating frail individuals, while their performance in distinguishing non-frail and pre-frail participants was close to the chance level. Furthermore, no significant differences were observed between IPAQ-SF and PASE across frailty categories, and both questionnaires showed moderate agreement with the FRAIL Scale when optimal cut-off points were applied.

Previous studies have reported considerable variations in the prevalence of frailty among older adults, depending on the setting and assessment method used. Community-based investigations have typically identified frailty rates ranging from 5 to 12.5% (23, 24), whereas studies recruiting participants from clinical or rehabilitation contexts have reported substantially higher rates, often between 30 and 48% (25, 26). In the present study, the observed frailty prevalence of 39.4% aligns closely with these clinical reports and should be interpreted in light of the study’s methodological purpose. This study aimed to evaluate the discriminative capacity of physical activity questionnaires across frailty categories rather than estimating population prevalence. Therefore, recruitment through outpatient and rehabilitation facilities represented an intentional design decision to ensure adequate representation of frail individuals for robust ROC-based analysis. This distribution is therefore considered an expected outcome of the sampling framework, not a selection bias.

The findings of current study are partly consistent with previous evidence emphasizing the strong association between low physical activity and frailty risk; however, they also highlight the limitations of self-reported physical activity questionnaires in diagnostic applications. For example, Zanotto et al. (19) reported that IPAQ-SF achieved a relatively high AUC (0.84) for frailty identification in a hemodialysis population, although this performance likely reflects sample-specific characteristics and reference standards rather than generalizable accuracy in community-dwelling older adults. Similarly, da Silva et al. (27) demonstrated that low levels of physical activity combined with prolonged sedentary time, as measured by the IPAQ, significantly increased the risk of frailty in community-dwelling older adults. These results support the epidemiological relevance of self-reported measures but underline their limited discriminative precision when used as stand-alone screening tools.

In our study, both the IPAQ-SF and PASE performed better in distinguishing frail from non-frail individuals than in differentiating adjacent categories, such as non-frail and pre-frail. This may be explained by the more pronounced reduction in physical activity typically observed among frail participants, which produces a clearer contrast. In contrast, pre-frail individuals represent a heterogeneous group, and their reported activity levels substantially overlap with those of the non-frail group. Such misclassification may also result from methodological issues: IPAQ-SF relies on metabolic equivalents that may poorly capture light-intensity daily activities, whereas PASE emphasizes specific activity domains, leading to differential sensitivities. Indeed, our data revealed significant disagreement between the two instruments, consistent with a prior report (28) showing variability in frailty prevalence depending on the choice of physical activity measure and cut-off values.

Measurement reliability also requires consideration. Previous research on patients with Parkinson’s disease reported that IPAQ-SF exhibited relatively low test–retest reliability, whereas PASE showed moderate reliability (29). Similar concerns were echoed by Theou et al. (18), who found that objective physical activity measures, such as accelerometry, explained greater variance in frailty indices than questionnaires. Moreover, Ma et al. (30) demonstrated that vigorous and moderate physical activity, but not light-intensity activity, were associated with reduced frailty risk in a large cohort, underscoring the importance of capturing meaningful activity levels. Additionally, Kim et al. (31) introduced a frailty phenotype questionnaire based on Fried’s criteria, which showed substantially higher diagnostic performance than general physical activity questionnaires, highlighting the potential advantage of purpose-built frailty tools.

From a clinical perspective, findings of the current study emphasize that although the IPAQ-SF and PASE are inexpensive, practical, and easily applicable in both clinical and community contexts, their diagnostic features remain suboptimal. Specifically, both instruments showed moderate sensitivity (IPAQ-SF: 71%; PASE: 79%) but only modest specificity (IPAQ-SF: 63%; PASE: 54%) in identifying frail individuals. While this suggests that they are relatively effective in capturing true frailty cases (high negative predictive value: 88–90%), their ability to exclude non-frail cases is limited, as reflected in their low positive predictive values (IPAQ-SF: 36%; PASE: 34%) and low-to-moderate overall accuracy (~60–65%). Furthermore, the diagnostic odds ratios (IPAQ-SF: 4.17; PASE: 4.33) indicate only modest discriminative utility, far from the levels expected of reliable screening tools. The differential patterns—slightly higher specificity for IPAQ-SF versus higher sensitivity for PASE—suggest that the two questionnaires capture somewhat distinct aspects of activity behavior, which may explain their moderate inter-instrument agreement (*κ* = 0.32). In practical terms, PASE may be preferred in contexts where the priority is to capture as many frail individuals as possible (screening and community-based settings), whereas IPAQ-SF may be more appropriate in scenarios where minimizing false positives is important, such as clinical trials or resource-limited healthcare environments.

The strengths of this study include the relatively large sample size, use of advanced ROC and multiclass analysis, and direct comparison of two widely used questionnaires against a standardized frailty scale. Importantly, to our knowledge, this is the first study in the literature to evaluate IPAQ-SF and PASE using a multiclass ROC framework alongside cut-off-based agreement statistics in older adults. This novel design allowed for a more comprehensive assessment of the diagnostic performance across frailty categories, distinguishing our findings from those of prior studies that mainly reported associations or prevalence estimates. However, owing to the cross-sectional design, the predictive validity of these instruments for incident frailty cannot be established. Furthermore, as both questionnaires depend on self-reported information, potential recall and social desirability bias may have led to an underestimation of between-group differences. Additionally, detailed sociodemographic variables such as education level and marital status were not collected, which may slightly limit the interpretation of population diversity and external generalizability. However, the available demographic and clinical data sufficiently characterized the target population for this study’s clinical context.

Future studies should validate these cut-off values in different populations and cultural settings, incorporate objective activity monitoring (e.g., accelerometry), and investigate whether combined assessment approaches enhance frailty screening and prediction. Such efforts will be critical for refining frailty identification strategies and guiding tailored preventive interventions.

In conclusion, IPAQ-SF and PASE demonstrated limited ability to discriminate frailty status in older adults, with moderate accuracy in identifying frail individuals. While these questionnaires remain practical tools for large-scale use, their application should be cautious and preferably combined with performance-based assessments to improve diagnostic precision in clinical and community settings.

## 5 Conclusion

In this study, neither the IPAQ-SF nor the PASE achieved clinically adequate discrimination of frailty when applied in isolation, and their overall performances were largely equivalent. Although IPAQ-SF demonstrated slightly higher specificity and PASE offered greater sensitivity, both instruments yielded only modest diagnostic odds ratios and low positive predictive values, limiting their practical utility as stand-alone screening tools. Accordingly, these questionnaires are better positioned as supplementary instruments within multicomponent frailty screening frameworks than as independent decision-making tools. Beyond its empirical findings, this study contributes by providing a comprehensive multi-metric evaluation of the IPAQ-SF and PASE within the same cohort and by outlining a methodological route toward more reliable frailty assessment strategies.

## Data Availability

The raw data supporting the conclusions of this article will be made available by the authors, without undue reservation.
