# Supplementary material for: Assessing frailty in older adults: discriminative properties of physical activity questionnaires
Source: Front Public Health. 2025 Dec 5;13:1702843. doi: 10.3389/fpubh.2025.1702843 (PMC12714643; doi:10.3389/fpubh.2025.1702843)
Supplement: Supplementary file 1 [file Data_Sheet_1.pdf]

STROBE Statement—Checklist of items that should be included in reports of ***cross-sectional studies***

	Item No	Recommendation
**Title and abstract**	1	(*a*) Title and Abstract explicitly mention *cross-sectional design* and diagnostic accuracy evaluation.
		(*b*) Abstract includes aim, participants (n=289), methods (ROC, DeLong test), and key results (AUCs, cut-offs)
**Introduction**		
Background/rationale	2	Introduction paragraphs 1–3 explain frailty background and the need for screening via Physical Activity questionnaires.
Objectives	3	End of Introduction clearly states objectives: to determine discriminative performance and cut-offs of IPAQ-SF and PASE.
**Methods**		
Study design	4	Methods section (2.1) states: "This cross-sectional study was conducted at…"
Setting	5	Methods (2.1) describe the setting (Hacettepe University), recruitment period (Dec 2024–Jun 2025).
Participants	6	Inclusion/exclusion criteria listed in method section. MMSE>24, ≥65 years, etc.
Variables	7	FRAIL Scale = outcome; IPAQ-SF & PASE = exposures; demographics = covariates. All defined in section 2.3.
Data sources/ measurement	8*	Each instrument's measurement method and Turkish validity references provided (Sağlam et al., Ayvat et al.).
Bias	9	Potential selection bias and recall bias discussed in Methods and Discussion. Recruitment and standardized data collection minimized bias.
Study size	10	N=289; post-hoc power analysis described results section.
Quantitative variables	11	Described in Statistical Analysis: continuous data summarized as mean ± SD; categorical as n(%); ROC with cut-offs.
Statistical methods	12	a) All analyses were performed in MATLAB R2023b. Descriptive statistics were presented as mean ± SD or n (%). Between-group discrimination was assessed using multiclass ROC analyses (one-vs-rest approach) with area under the curve (AUC), sensitivity, specificity, predictive values, and diagnostic odds ratios. The DeLong and bootstrap methods were used to estimate 95% confidence intervals. Confounding control was not required as this was a diagnostic accuracy study without predictor–outcome adjustment.
		(*b*) Subgroup ROC analyses were performed by sex (male vs. female) and by age groups (65–74 vs. ≥ 75 years). Results are presented in Supplementary Table S3, showing similar AUC trends without statistically significant interactions (DeLong p > 0.05).
		(*c*) Missing data were minimal (< 2%) and handled through listwise deletion. The final sample included 289 complete observations across all variables.
		(*d*) The study employed convenience and snowball sampling from community and outpatient settings. Analyses did not require weighting or stratification, as all participants were treated as a single analytical group.
		(*e*) Sensitivity of frailty discrimination was examined through both ROC and precision–recall (PR) curve analyses. Diagnostic indices were compared across alternative cut-off thresholds using the Youden index to ensure robustness of results.
**Results**		
Participants	13*	(a) A total of 312 older adults were initially approached through community advertisements and outpatient clinics.After eligibility screening, 289 participants met inclusion criteria (≥65 years, MMSE > 24, no severe neuropsychiatric or motor impairments) and provided written informed consent.All 289 participants completed the full assessment battery, including demographic data, FRAIL Scale, IPAQ-SF, and PASE questionnaires.There were no dropouts or missing outcome data, and therefore all 289 individuals were included in the final statistical analyses.Because the study was cross-sectional with a single assessment session, no follow-up stage or attrition occurred; thus, a flow diagram was not deemed necessary.
		**Did not meet inclusion criteria (n = 12):** MMSE ≤ 24 and/or major neurological/psychiatric conditions.<br>**Did not consent / declined to participate (n = 11):** time or transportation constraints.

		A flow diagram was not included, as the study was cross-sectional with a single assessment session and no follow-up or participant attrition.All 289 eligible participants who met inclusion criteria and provided consent were included in the final analysis.
Descriptive data	14*	(a) Table 1 includes demographics and clinical characteristics; no missing data reported.
		(b) Table 1 includes demographics and clinical characteristics; no missing data reported.
Outcome data	15*	Frailty status, determined using the FRAIL Scale, was categorized as non-frail (n = 90, 31.1%), pre-frail (n = 85, 29.4%), and frail (n = 114, 39.4%).These outcome categories were used in all receiver operating characteristic (ROC) analyses for discriminative performance. Summary measures of diagnostic accuracy (AUCs, sensitivity, specificity, predictive values, and diagnostic odds ratios) for both IPAQ-SF and PASE are presented in Tables 2–4 and visually depicted in Figures 1–2.
Main results	16	(*a*) All reported estimates were unadjusted, as the analyses focused on diagnostic performance rather than association modeling.Receiver Operating Characteristic (ROC) analyses were used to calculate AUC values, sensitivity, specificity, positive/negative predictive values, accuracy, and diagnostic odds ratios for each frailty category.Precision of estimates was provided as 95% confidence intervals, calculated using both the DeLong and bootstrap methods.No confounder adjustment was performed because the study did not include independent predictors or covariate modeling; the goal was purely discriminative evaluation of IPAQ-SF and PASE performance.
		(*b*) Continuous total scores from the IPAQ-SF (MET-min/week) and PASE were dichotomized into "frail" versus "non-frail" classifications based on optimal ROC-derived cut-off points using the Youden index.The optimal thresholds were ≤ 322.5 MET-min/week for the IPAQ-SF and ≤ 63.6 points for the PASE.These category boundaries were used to calculate diagnostic indices and cross-tabulation agreement with the FRAIL Scale (see Table 4).
		(*c*) Not Applicable
Other analyses	17	Additional analyses were conducted to evaluate the robustness and generalizability of results. Subgroup analyses were performed by sex (male vs. female) and age groups (<71 vs. ≥71 years), revealing similar AUC patterns without statistically significant differences (DeLong p > 0.05). These results are presented in Supplementary 2. Sensitivity analyses included comparing diagnostic indices across alternative ROC cut-off thresholds (using the Youden index) and evaluating model robustness via precision–recall (PR) curves. Both analyses confirmed that IPAQ-SF and PASE exhibited consistent, moderate discriminative ability for identifying frail individuals.
**Discussion**		
Key results	18	The Discussion section begins with a concise summary of the main findings in direct relation to the study objectives. Both physical activity questionnaires (IPAQ-SF and PASE) demonstrated limited ability to discriminate frailty levels, showing fair accuracy for identifying frail individuals but poor discrimination between non-frail and pre-frail groups. No statistically significant differences were found between instruments, aligning with the study's objective to compare their diagnostic performance and optimal cut-offs for frailty screening in older adults.
Limitations	19	This study has several limitations that should be acknowledged. First, its cross-sectional design precludes conclusions about predictive validity or causal relationships between physical activity and frailty progression. Second, the recruitment strategy, which combined outpatient clinics and community-based participants, may have introduced selection bias, resulting in a higher frailty prevalence (39.4%) than expected in general community samples. Third, both physical activity questionnaires (IPAQ-SF and PASE) rely on self-reported data, which are prone to recall and social desirability bias, potentially underestimating discriminative performance. Moreover, the sample had limited demographic diversity beyond age and sex, reducing the generalizability of findings to more heterogeneous populations. Finally, the absence of objective or performance-based physical activity measures (e.g., accelerometry) restricted the ability to validate self-reports directly. Taken together, these biases may have attenuated between-group differences and led to conservative estimates of diagnostic accuracy. Nevertheless, the use of validated Turkish versions of the instruments, standardized data collection procedures, and supplementary subgroup analyses contribute to the robustness and internal validity of the reported results.

Interpretation	20	The results of this study should be interpreted in light of the acknowledged limitations, yet several methodological strengths also enhance its validity. The study included a relatively large sample of community-dwelling older adults, employed advanced ROC and multiclass analyses, and directly compared two widely used self-report physical activity questionnaires (IPAQ-SF and PASE) against a standardized frailty scale. To our knowledge, this is the first study to apply a multiclass ROC framework and cut-off–based agreement statistics to evaluate frailty discrimination in older adults. This novel analytic design enabled a more comprehensive assessment of diagnostic performance across frailty categories, distinguishing the present findings from prior research focused primarily on associations or prevalence. Future studies should validate the proposed cut-off values in diverse populations and cultural settings, integrate objective physical activity monitoring (e.g., accelerometry), and explore combined assessment models to enhance the predictive precision of frailty screening tools.
Generalisability	21	The findings of this study are primarily generalizable to clinically evaluated, community-dwelling older adults, reflecting the intended target population for frailty screening. Recruitment from both community and outpatient settings was an intentional design choice to capture a realistic spectrum of health and functional status among older adults who are most likely to undergo frailty assessment in clinical or preventive contexts. Therefore, the sample composition aligns with the study's applied objectives rather than representing a bias. While the observed frailty prevalence (39.4%) may differ from large-scale epidemiological estimates, it appropriately reflects the clinical context in which such questionnaires are typically used. The consistent discriminative trends across sex and age subgroups support the external validity and applicability of the proposed cut-off values within similar healthcare and cultural settings. Nonetheless, further validation in population-based and longitudinal cohorts would strengthen generalizability to broader aging populations.
**Other information**		
Funding	22	This research did not receive any external funding from public, commercial, or not-for-profit agencies.

*Give information separately for exposed and unexposed groups.

Note: An Explanation and Elaboration article discusses each checklist item and gives methodological background and published examples of transparent reporting. The STROBE checklist is best used in conjunction with this article (freely available on the Web sites of PLoS Medicine at http://www.plosmedicine.org/, Annals of Internal Medicine at http://www.annals.org/, and Epidemiology at http://www.epidem.com/). Information on the STROBE Initiative is available at www.strobe-statement.org.
