# Supplementary material for: Assessing frailty in older adults: discriminative properties of physical activity questionnaires
Source: Front Public Health. 2025 Dec 5;13:1702843. doi: 10.3389/fpubh.2025.1702843 (PMC12714643; doi:10.3389/fpubh.2025.1702843)
Supplement: Supplementary file 2 [file Data_Sheet_2.pdf]

Subgroup analyses revealed that the diagnostic performance of the IPAQ-SF for frailty classification varied across sex and age groups. Among females, the AUC values ranged from 0.34 (pre-frail) to 0.66 (frail), whereas among males they ranged from 0.36 (non-frail) to 0.65 (frail). Similar patterns were observed across age categories, with higher discriminatory capacity in the frail subgroup (AUC = 0.75 for < 71 years; AUC = 0.60 for ≥ 71 years). These findings indicate that physical activity levels measured by IPAQ-SF show modest accuracy for distinguishing frailty status, particularly within older and frail individuals (Table 1).

Table 1. Diagnostic performance of IPAQ-SF for frailty classification: subgroup ROC analysis results.

**Sub-Group**	**Comparison**	**AUC_95CI**	**AUCB_95CI**	**Optimal_Cutoff**	**Sensitivity**	**Specificity**	**Accuracy**	**PPV**	**NPV**	**F1_score**	**MCC**	**DOR**
Female	Non-frail (n=56)	0.54 (0.40–0.68)	0.54 (0.39–0.68)	346.5	0.55	0.68	0.66	0.27	0.88	0.36	0.18	2.55
	Pre-frail (n=36)	0.34 (0.21–0.46)	0.34 (0.22–0.47)	2199	0.86	0.14	0.22	0.11	0.89	0.20	0.00	1.01
	Frail (n=33)	0.66 (0.53–0.80)	0.66 (0.52–0.79)	693	0.94	0.44	0.50	0.21	0.98	0.34	0.27	12.33
Male	Non-frail (n=34)	0.36 (0.24–0.48)	0.36 (0.25–0.48)	0	0.00	1.00	0.68	0.09	0.83	0.00	-0.09	0.50
	Pre-frail (n=49)	0.42 (0.31–0.53)	0.42 (0.31–0.53)	1386	1.00	0.11	0.24	0.16	1.00	0.28	0.13	2.88
	Frail (n=81)	0.65 (0.57–0.74)	0.65 (0.56–0.73)	445.5	0.90	0.49	0.61	0.43	0.92	0.58	0.36	8.35
Age < 71	Non-frail (n=58)	0.46 (0.33–0.60)	0.46 (0.32–0.60)	148.5	0.21	0.84	0.74	0.22	0.84	0.21	0.05	1.43
	Pre-frail (n=44)	0.47 (0.35–0.60)	0.47 (0.35–0.60)	1188	0.75	0.33	0.39	0.16	0.89	0.26	0.06	1.50
	Frail (n=38)	0.75 (0.66–0.85)	0.75 (0.65–0.84)	322.5	0.71	0.78	0.76	0.48	0.90	0.57	0.43	8.66
Age ≥ 71	Non-frail (n=32)	0.41 (0.30–0.52)	0.41 (0.30–0.52)	358	0.61	0.42	0.45	0.16	0.85	0.25	0.02	1.13
	Pre-frail (n=41)	0.34 (0.21–0.46)	0.34 (0.22–0.46)	2079	1.00	0.10	0.21	0.13	1.00	0.23	0.11	1.98
	Frail (n=76)	0.60 (0.50–0.69)	0.60 (0.50–0.69)	264	0.71	0.49	0.54	0.30	0.85	0.42	0.18	2.41

Note. Age classified by the sample median (≤ 71 vs > 71 years). AUC—area under the curve; AUCB—area under the curve with 5000 Bootstrap; CI—confidence interval; PPV—positive predictive value; NPV—negative predictive value; MCC—Matthew's correlation coefficient; DOR—diagnostic odds ratio.

Comparable subgroup analyses for the PASE demonstrated a similar trend. The AUC values ranged from 0.45 to 0.64 among females and from 0.34 to 0.65 among males. In age-based groups, classification performance was slightly better in participants aged < 71 years (AUC = 0.70 for frail) compared to those ≥ 71 years (AUC = 0.59 for frail). Although the overall discriminatory ability of PASE was limited, the instrument showed fair accuracy for identifying frail individuals, consistent with the IPAQ-SF results. Complete diagnostic indices including sensitivity, specificity, predictive values, and diagnostic odds ratios are summarized in Table 2.

Table 2. Diagnostic performance of PASE for frailty classification: subgroup ROC analysis results.

Sub-Group	Comparison	AUC_95CI	AUCB_95CI	Optimal_Cutoff	Sensitivity	Specificity	Accuracy	PPV	NPV	F1_score	MCC	DOR
Female	Non-frail (n=56)	0.45 (0.30–0.59)	0.45 (0.30–0.59)	6.4	0.18	0.88	0.76	0.25	0.83	0.21	0.07	1.69
	Pre-frail (n=36)	0.48 (0.30–0.67)	0.48 (0.30–0.67)	37.31	0.36	0.79	0.74	0.18	0.91	0.24	0.11	2.13
	Frail (n=33)	0.64 (0.52–0.77)	0.64 (0.52–0.76)	63.6	0.76	0.58	0.61	0.22	0.94	0.35	0.24	4.55
Male	Non-frail	0.34 (0.21–0.47)	0.34 (0.22–0.47)	14.03	0.20	0.85	0.75	0.19	0.86	0.20	0.05	1.40
	Pre-frail	0.48 (0.36–0.59)	0.48 (0.36–0.59)	61.23	0.67	0.44	0.47	0.17	0.88	0.27	0.07	1.54
	Frail	0.65 (0.55–0.74)	0.65 (0.55–0.74)	55.5	0.76	0.61	0.65	0.45	0.85	0.56	0.33	4.80
Age < 71	Non-frail (n=58)	0.38 (0.24–0.53)	0.38 (0.25–0.53)	7.31	0.17	0.92	0.79	0.31	0.84	0.22	0.12	2.38
	Pre-frail (n=44)	0.55 (0.41–0.69)	0.55 (0.41–0.69)	61.23	0.60	0.68	0.67	0.24	0.91	0.34	0.21	3.24
	Frail (n=38)	0.70 (0.60–0.81)	0.70 (0.59–0.80)	62.09	0.71	0.69	0.69	0.39	0.89	0.51	0.34	5.39
Age ≥ 71	Non-frail (n=32)	0.38 (0.25–0.52)	0.38 (0.26–0.52)	14.03	0.22	0.83	0.73	0.19	0.85	0.20	0.04	1.31
	Pre-frail (n=41)	0.44 (0.30–0.58)	0.44 (0.30–0.58)	173.15	1.00	0.03	0.15	0.12	1.00	0.22	0.06	0.57
	Frail (n=76)	0.59 (0.50–0.69)	0.59 (0.49–0.69)	55.5	0.80	0.54	0.60	0.35	0.90	0.49	0.29	4.77

Note. Age classified by the sample median (≤ 71 vs > 71 years). AUC—area under the curve; AUCB—area under the curve with 5000 Bootstrap; CI—confidence interval; PPV—positive predictive value; NPV—negative predictive value; MCC—Matthew's correlation coefficient; DOR—diagnostic odds ratio.

The multiclass ROC comparison between IPAQ-SF and PASE showed no statistically significant differences in AUC values across sex or age strata. For example, among females, AUCs were nearly identical (macro AUC = 0.51 vs 0.52), and similar patterns were observed in males (0.48 vs 0.49) and in younger (0.56 vs 0.55) and older groups (0.45 vs 0.47). DeLong's test confirmed the absence of significant differences between instruments in any frailty category (all $p > 0.05$). These results suggest that both physical activity questionnaires exhibit comparable diagnostic capacity for classifying frailty (Table 3).

Table 3. Multiclass ROC analysis and comparisons of AUC values between IPAQ-SF and PASE.

Group	Performance_Metrics	IPAQ_SF	PASE	z	p
Female	Macro Average AUC	0.51	0.52		
	Micro Average AUC	0.52	0.52		
	Non-frail	0.54	0.45	0.95	0.34
	Pre-frail	0.34	0.48	-1.51	0.13
	Frail	0.66	0.64	0.30	0.76
	Micro (pooled OvR)	0.52	0.52	0.09	0.93
Male	Macro Average AUC	0.48	0.49		
	Micro Average AUC	0.51	0.51		
	Non-frail	0.36	0.34	0.25	0.80
	Pre-frail	0.42	0.48	-0.83	0.41
	Frail	0.65	0.65	0.13	0.90
	Micro (pooled OvR)	0.51	0.51	-0.22	0.83
Age < 71	Macro Average AUC	0.56	0.55		
	Micro Average AUC	0.58	0.56		
	Non-frail	0.46	0.38	0.78	0.44
	Pre-frail	0.47	0.55	-0.90	0.37
	Frail	0.75	0.70	0.90	0.37
	Micro (pooled OvR)	0.58	0.56	0.53	0.59
Age ≥ 71	Macro Average AUC	0.45	0.47		
	Micro Average AUC	0.47	0.49		

	Non-frail	0.41	0.38	0.33	0.74
	Pre-frail	0.34	0.44	-1.35	0.18
	Frail	0.60	0.59	0.03	0.98
	Micro (pooled OvR)	0.47	0.49	-0.47	0.64

*The statistical significance between the AUC values of IPAQ-SF and PASE was compared using the DeLong test. OvR: one vs rest.
